# RE: Conceptual competence in psychiatric training: building a culture of conceptual inquiry

**DOI:** 10.1192/bjb.2024.53

**Published:** 2024-08

**Authors:** Alistair Stewart

**Affiliations:** Consultant Psychiatrist, Fairfield General Hospital, Bury, UK. Email: alistair.stewart@nhs.net

*Talking philosophy*

Any proposal to encourage philosophical enquiry in a field such as psychiatry inevitably raises further philosophical questions. That is in the nature of these things. In any case, the detailed arguments presented by Dr Aftab and colleagues for making philosophy integral to the training of psychiatrists are heartily welcome, as are all similar initiatives. Let a hundred flowers and entry points bloom! There is, of course, a tension between wanting to open the widest and most searching type of enquiry and the compromises which may be needed to secure the place of philosophy in formal training and the curriculum. Aftab and colleagues leave to one side the tricky question of how openness to, awareness of and indeed ‘competence’ in philosophical matters may be assessed. Wilfred Sellars offered the following succinct and satisfyingly vague and general formulation of the aim of philosophy:^1^ *to understand how things in the broadest possible sense of the term hang together in the broadest possible sense of the term*. In that spirit, I think we should be wary of delineating the task too narrowly. Conceptual competence, as an analogy to clinical competence, is unhelpful as it implies the acquisition of a set of skills rather than an attitude of curiosity and a willingness to tolerate uncertainty. How to transmit those things is certainly a challenge. For example, I suspect that many doctors, myself included, could do with learning a little more humility, but that is likely to require more than an understanding of concepts. In addition, while getting people to interrogate the problem-ridden concepts which abound in psychiatry, it may not be the most helpful thing to introduce yet more problem-ridden concepts, such as ‘teaching conceptual competence’, or to limit the possible types of opportunity for teaching to four. There are surely few scenarios in psychiatry or medicine, or indeed in everyday life, which do not provide material for philosophical reflection. This is the perspective we need to foster in our trainees and indeed in ourselves, and we will achieve it best through dialogue and, above all, by making the experience interesting and fun! Aftab et al are refreshingly honest in acknowledging the risk that the term ‘conceptual competence’ introduces ‘managerial language’. However, the plea that we are ‘speaking the language of our time’ worries me. Clearly, in one sense, we do need to speak in contemporary terms if we are to communicate effectively with the people we want to persuade, including in philosophy – as one eminent philosopher has put it, ‘we have to roll our own’. In another sense though, the role of philosophy is precisely to challenge the language of our time, not to subordinate itself to it – otherwise we are starting out with one hand tied behind our back. It was not for nothing that Nietzsche entitled his 1876 set of essays *Untimely Meditations*.^2^
